# Malaria and Colonialism in Korea, *c.*1876–*c.*1945

**DOI:** 10.1093/shm/hkv110

**Published:** 2015-10-12

**Authors:** Jeong-Ran Kim

**Keywords:** malaria, Korea, Japanese imperialism, colonial medicine, endemic diseases

## Abstract

This article considers the problem of malaria in the Korean peninsula from 1876 to 1945, focusing particularly on the impact of Japanese colonial rule. One aspect which receives special attention is malaria in urban contexts. The relationship between malaria and urbanisation is shown to be extremely complex, fluctuating regardless of specific interventions against the disease. In rural and urban areas, Japanese antimalarial measures concentrated on military garrisons, at the expense of both civilian settlers and Koreans. However, it was Koreans who bore the brunt of the malaria problem, which was exacerbated in many areas by agricultural and industrial development and, ultimately, by the war regime introduced from 1938. The worsening of the malaria burden in the final years of Japanese rule left a legacy which lasted long after independence.

Malaria had long been regarded as a problem in Korea but colonial ‘development’ policies led to dramatic fluctuations in its incidence among Koreans. The disease also took its toll on the Japanese, being a perennial cause of illness among troops and civilians. In both respects, the experience of Japanese colonialism in Korea was similar to that of territories acquired by the European powers in other parts of Asia. In British India, for example, malaria was the greatest cause of mortality among Indian civilians and one of the largest causes of sickness in British garrisons.^1^ But the ways in which the Japanese dealt with malaria in Korea differed from the interventions of the European powers and also from those of Japan in its other colonial territories, notably Taiwan. This article seeks to explain these differences against the background of Korea's changing epidemiological situation.

First, it is necessary to consider what ‘malaria’ was. Before the late 1890s and Ronald Ross's discovery of the role of the *Anopheles* mosquito as the vector in malaria, Western doctors used the term ‘malaria’ principally to refer to diseases which arose from swamps and other poorly drained land. The term did not necessarily denote a specific disease, despite Laveran's identification of the malaria parasite (*Plasmodium*) in 1880. There was little agreement about how the parasite was conveyed and it took many years to convince the majority of doctors that it was even the cause of ‘malarious fevers’. For many, it remained an article of faith that these diseases—alternatively termed ‘intermittent’ or ‘remittent’ fevers—were caused by effluvia from rotting matter. This belief was related to the sanitary concerns of the time, which saw filth as the principal cause of many diseases.^2^

Malaria, then, was not simply a climatic disease but was increasingly associated with the lack of civilisation. European countries and colonial regimes therefore began to drain marshes in and around cities and the discovery of the mosquito vector gave a massive boost to this endeavour.^3^ Anti-malarial measures gradually became a prominent part of the imperial ‘civilising mission’; in part because of the international attention which was given to epidemics such as that in the Punjab in 1908, in which over one-quarter of a million people died. Serious outbreaks of malaria also occurred among migrant labourers employed on reclamation works, dockyards and similar enterprises in booming cities.^4^ Migrant labourers additionally brought malaria into non-malarial areas. These included not only cities but also rural areas in which plantation and other forms of cultivation were expanding. In Sarawak, for example, malaria became particularly severe in districts which received immigrants from China and Johore.^5^

These problems were not confined to the tropics or, indeed, to the colonies of Western powers. A latecomer to the imperial race, Japan soon found that malaria was one of its greatest foes following the invasion of Taiwan in the 1870s. Until the 1890s, malaria mortality was far higher for Japanese immigrants in Taiwan than for the local Taiwanese.^6^ To protect Japanese settlers and soldiers, and to eliminate obstacles to natural resource development, the colonial government began to take anti-malarial measures which were ‘imposed from above’ rather than ‘evolved from below’, through interaction with colonised people. From the 1920s, anti-malaria efforts were also deemed part of the broader civilising and modernising mission of the Japanese colonial government.^7^ However, as Ya-wen Ku has pointed out, most malaria epidemics were caused by the government's efforts to develop the colony, producing massive environmental change.^8^

The situation in the Korean peninsula was similar. Even though Korea has a temperate climate, with four distinct seasons, malaria was widespread in much of the peninsula. The most common form of malaria in Korea was *Plasmodium vivax*, which was very widespread but less deadly than the *P. falciparum* form of malaria commonly found in Taiwan. It was a persistent problem of long standing. Records of a disease which was almost certainly malaria, *hakjil*, appear from the Goryeo period (918–1392), being frequently reported in the *Annals* of that and subsequent dynasties.

When the Japanese began to appear in large numbers, after the opening of Korean ports in 1876, they suffered badly from malaria and several other diseases which were endemic to the country. The same was true of new arrivals from China and the West. One of the biggest problems for the Japanese in Korea was malaria among young adult and middle-aged people, including troops but, by contrast to many other colonial situations, mortality from malaria was highest among Koreans. Although, the colonial government began to invest in malaria prevention from 1928, it paid little attention to malaria by comparison to other acute infectious diseases and some endemic diseases such as pulmonary distoma and leprosy, even though they caused far less morbidity and mortality.^9^ This neglect stands in stark contrast to the authoritarian interventions made by the Japanese in Taiwan and even from the less consistent interventions of European colonial powers.

The colonial government's neglect of malaria is mirrored by that of historians. Although Wataru Iijima has written on the problem of malaria in the Japanese empire, his principal focus lies elsewhere—on Taiwan. Iijima has pointed out that the control of malaria enabled the Japanese to penetrate local communities. He also shows how the Japanese authorities and doctors developed tropical medicine in order to deal with the severe form of malaria found on the island, and how they later transplanted this knowledge to Okinawa. The new discipline thus became central to imperial medicine and was a driving force of Japanese expansion into the Asian continent. Korea, by contrast, receives little attention in Iijima's work and is discussed only in relation to a handful of Japanese experts. The burden of malaria on the civilian population is ignored.^10^ One of the few historical studies concentrating on malaria in Korea is that by In-Sok Yeo. While it provides a valuable overview of key developments in research, it does not make a detailed assessment of the colonial government's anti-malaria measures or of the relationship between malaria and colonialism.^11^

This article aims to fill this gap by evaluating the impact of Japanese rule on the malaria situation in Korea, from the time of the initial settlements in 1876 to the end of colonial rule in 1945. One aspect which receives special attention is malaria in urban contexts. Although it is generally assumed that urbanisation tends to diminish malaria, it will be shown that the relationship between its incidence and urban development was extremely complex, sometimes tending to increase cases and sometimes to reduce them. As in rural areas, malaria was exquisitely sensitive to the nature of economic activity, as well as to the movement of migrant labourers. This article tracks these fluctuations in relation to colonial development regimes and considers the response of local and imperial governments. It ends by examining the legacy of the Japanese war regime in Korea, which influenced the malaria situation for many years after the nation's independence.

## ‘Another permanent abode of malaria’: pre-colonial Korea

Malaria was a very common disease among Koreans, whether ordinary civilians or members of the royal family. In the summer of 1421, for example, Prince Yang-Nyeong was affected by a disease which was almost certainly malaria and the renowned monarch King Sejong sent his court physician to tend his son.^12^ According to Hwang-Hyeon, an eminent late Choseon era (1392–1910) writer, Koreans were in constant fear of malaria due to high mortality from the disease and the fact that it weakened people severely, leaving them unable to work.^13^ After the introduction of quinine, however, many of the lives that would formerly have been lost to malaria were saved.^14^ A missionary doctor, H. N. Allen, first director of Jejung Hospital which was the first Westernized hospital in Korea built by the Korean government in 1885, was one of those who regularly used quinine to treat malaria patients.^15^

The extent of the malaria problem in Korea is also shown by the writings of Japanese doctors from the time of the opening of Korean ports. For example, Dr Koike, who was an army medical doctor and the director of a state hospital (serving from 1883 to 1885) in the Japanese settlement in Busan, Saisei Iin, explained that after diseases of the digestive system, malaria was the second largest cause of admissions among Korean patients.^16^ At that time, Japanese doctors believed that malaria was caused by the presence of putrid effluvia in the atmosphere, like most Western doctors.^17^ For example, a doctor in Kōchi Prefecture argued that malaria was caused by poisonous gases arising from filth; therefore sanitary reform was needed to prevent it.^18^ Dr Koike also pointed out that malaria was prevalent in swampy land, such as that around the port of Incheon.^19^

After Korea's ports were opened by Japan, in 1876, many Japanese people flocked there to make money. As the first treaty port in Korea, the south-eastern port of Busan became, to all intents and purposes, an imperial city even before Japan became a colonial power in a formal sense. Other settlements soon followed. In the north-east, Wônsan, which is now located in North Korea, was opened in 1880 to prevent Russia's movement southward. The port of Incheon, which is close to Seoul, was opened in 1883. There, foreign influence was not confined only to the Japanese, for Incheon was a gateway for goods and people moving between Korea, Japan and China. In 1882, China also forced Korea to make a trade treaty, with the object of containing Japanese influence and securing its dominant position in the peninsula. With the same object in mind, China permitted the Korean government to make commercial treaties with the USA and, the year after, with Britain.^20^ After making these treaties, not only Japanese but Chinese and Westerners began to live in Korea and to witness or succumb to the so-called ‘Korean fever’.^21^

However, it is important to consider the malaria problem in context, as the comparative effort to control the various diseases in Korea is instructive. Japanese settlers and soldiers suffered a variety of infectious diseases, such as cholera, dysentery and typhoid, largely due to the lack of an adequate water supply in their settlements. These diseases were also common in Japan and may even have been brought to Korea. In 1912, a Japanese doctor of the Busan Hospital for Infectious Diseases pointed out that ‘dysentery among the Japanese settlers is bacillary dysentery which was then common in Japan. Most Koreans, by contrast, were infected with amoebic dysentery.’ He thus concluded that dysentery among the settlers was related to interchange between the settlement and homeland.^22^ Some areas of Japan were malarious, too. The so-called ‘benign tertian malaria’ (caused by the *Plasmodium vivax* parasite) was scattered throughout the country, particularly, Kōchi, Shiga, Fukui, Shizuoka, Tochigi, Niigata, Gunma, Aomori Prefecture and Kyoto, where it occurred principally in low-lying damp districts which favoured the propagation of the *Anopheles* mosquito. ‘Quartan’ malaria (a relatively mild form of the disease caused by *P. malariae*) and falciparum malaria were also found in the Okinawa Prefecture.^23^ But during this period, most Japanese people in Korea came from areas in which malaria was less prevalent, such as Nagasaki, Yamaguchi, Ōita and Fukuoka Prefecture. In 1896, these four Prefectures accounted for 71.7 per cent of the Japanese population in Korea.^24^ Malaria was therefore a relatively new disease for most Japanese settlers and few had any immunity to it. In 1887, for example, out of a total of 2,006 Japanese settlers of Busan, 493 people were infected with malaria.^25^ The year before, cholera had spread from Japan to Korea and many Koreans and Japanese were affected. By the following year, cholera disappeared but a malaria epidemic occurred, being particularly severe in Busan. Malaria remained a serious problem in Wônsan, too. In 1892, for example, 234 of the 2,270 patients treated at the public hospital in the Japanese settlement, were admitted with malaria.^26^ Japanese garrisons in Korea were equally affected. In 1897, soldiers of the garrison in Seoul were suffering from dysentery, pleurisy and malaria, amongst other diseases.^27^ The same year, the Japanese soldiers in Busan were infected by malaria but their symptoms were relatively mild.^28^

Westerners living in Korea experienced similar problems. A British medical practitioner explained that just after the treaty was signed between Korea and Britain, half a dozen cases of intermittent fever had occurred among the British servants and workers in Seoul and Incheon. Other types of fever were also apparently very common in Incheon and residents often suffered from a variety of ailments simultaneously.^29^ For example, in July 1887, Mr Watters, H. M. Consul General in Seoul sent a letter to the British envoy in Peking, requesting two months' leave of absence that autumn as he was suffering from fever and extreme debility, complicated by beriberi. He added that it was quite impossible to go on living there in houses which were ‘a permanent abode of fever and poisons malaria’.^30^ In 1897, a Russian naval officer who visited Incheon to find land on which to construct a naval hospital mentioned that the climate of Incheon was good but many diseases were prevalent, including malaria. Thus he recommended Wôlmi island in Incheon, which appeared to be free from malaria. He explained that some Western doctors in Seoul moved from Korean houses which were located in the lower part of this city to European style houses on the hillside. This had enabled them to escape from the danger of malaria.^31^ Oliver R. Avison—a missionary doctor who built the subsequently famous Severance Hospital in 1904—also mentioned the problem of malaria in Korea. He came to Korea in July 1893 and became the director of Jejung hospital. Before the mosquito vector theory of malaria was known, he recommended Westerners to build houses on hillsides and to have their bedrooms upstairs because he thought malaria was caused by bad air emanating from marshland.^32^

After the 1900s, the cases of malaria decreased in some areas, especially where the Japanese settlements were located. The commander of the Japanese garrison in Busan reported that while malaria was formerly one of the most severe endemics in the area, it had all but disappeared.^33^ The Japanese vice consul in Wônsan also mentioned that just after the port was opened, about 80–90 per cent of patients were suffering from malaria but that cases had been falling gradually and there were now only a few.^34^ This decline did not occur because the Japanese authorities had intervened to prevent malaria. No specific regulations against the disease had been devised but general sanitary reforms in Japanese settlements during the pre-colonial period probably contributed to it. In the Japanese settlement in Busan, in June 1880, a sanitary conference was held, sponsored by the consular office, to discuss the enactment of rules for the prevention of epidemics and the public health of the settlement, in view of a cholera epidemic the previous year. As a result, the *Rules for the Prevention of Cholera* were produced in 1880. These provided for the cleaning of the shores, lavatories and dwellings, as well as the removal of ill-smelling things.^35^ Thereafter, the Japanese consul of Busan enacted related rules and laws and implemented sanitary reforms, including the improvement of roads, the building of sewerage systems and the supply of clean drinking water.

From the start of the Russo-Japanese war, Japanese imperialism began to spread further into the Asian continent. Preparing for the war, Japan acquired the right from the Korean government to construct a railway between Busan and Seoul (the so-called Gyeongbu Cheoldoseon). In the midst of war, the railway was opened to traffic in January 1905 and, in the same year, the Kanpu Ferry which plied between Busan and Shimonoseki, began to operate, making Busan the bridgehead for Japanese expansion into Asia. In view of its rising importance, the Japanese resolved to transform Busan into a modern port city and the sanitary implications of this had to be recognised if it was to function effectively.^36^ In 1906, in an attempt to improve the situation, the settlers in Busan, as well as other areas in Korea and China, were reorganised into corporate bodies (the so-called *Kyoryūmindan*) capable of supporting national policies through urban renewal.^37^ These sanitary reforms seemed to reduce malaria in Busan, especially in the Japanese settlement and its surrounding new town, which was created for Japanese immigrants. This mirrors developments in Japan's first colony, Taiwan, particularly in Taipei, where a remarkable reduction in malaria took place after urban sanitary reforms were carried out, including sewerage systems and improvements to housing.^38^

The reduction of malaria in some cities controlled by the Japanese followed much the same pattern as in Western colonies in Southeast Asia. In Dutch Java, British Malaya and French Indochina, malaria became rare in cities but remained prevalent in rural areas. A Japanese naval doctor explained that the reason was that Western authorities carried out urban sanitary reforms for their people who lived in the cities.^39^ However, it was not simply progressive urbanisation but rising living standards and educational provisions in the cities, which were important in reducing mosquito populations there. In all these respects, rural areas lagged behind.^40^ In Italy's case, until the early twentieth century, even public health clinics were unequal to the task of providing medical attention to the rural poor. For this reason, the anti-malarial campaign established a new institution, the rural health station, in the countryside.^41^ But these were general patterns rather than universal laws. In some cases, urban development caused overpopulation and unsanitary conditions, which led to cases of malaria increasing. In rapidly expanding cities such as the great Indian port of Bombay, malaria cases tended to fluctuate, depending on the vigour with which anti-malaria measures were enforced.^42^

## Annexation

After its victory in the Russo-Japanese war, Japan organised the Residency-General in Korea in 1906. As a result, the Korean government was deprived of its right to conduct diplomacy independently of Japan. In 1907, Hirobumi Itō, who was the first Resident-General, took the crown from the Korean king, Gojong, and seized power over internal affairs from the Korean government. Finally, in August 1910, Japan annexed Korea forcibly.

Even before the annexation, since 1905, the state hospital and Western style medical school, which both were built in 1899, came under Japanese control, which frustrated some recent initiatives taken by the Korean government to reform medical care and public health.^43^ In June 1910, the police system of the Korean government was absorbed into that of the Residency-General. Afterwards, the sanitation department was incorporated into the police system in order to manage the sanitary administration of Seoul and surrounding areas. Following the annexation, the Government-General centralized the sanitary police system in Korea and, in the following year, the sanitary police were given control over all aspects of administration, except the management of the state and charitable hospitals which were directed by the Japanese military doctors.^44^

**Fig. 1:**
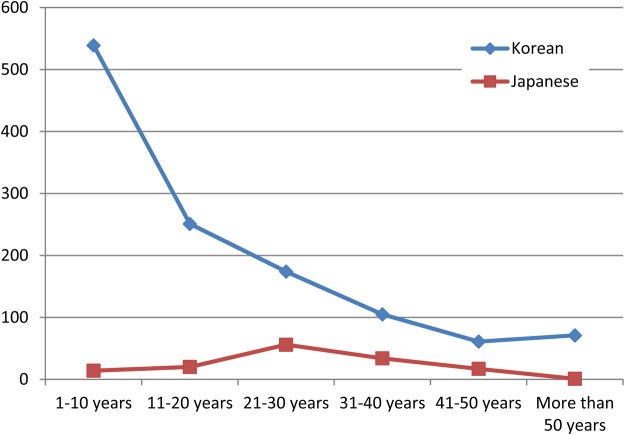
Number of malaria patients in the main state hospitals in 1911. *Source*: Army Medical Officer, *Chōsenjin no Ishokujū oyobi Sono Ta no Eisei* (Seoul: Unknown publisher, 1915), 156.

The Government-General's sanitary policies focused on acute infectious diseases and it was for this purpose, in June 1915, that the colonial government enacted the *Laws to Prevent Infectious Diseases of Korea* (*Chōsen Densenbyō Yobōrei*). However, the high morbidity of Japanese settlers continued well into the colonial period. In 1922, for example, 1,170 Japanese settlers were affected by dysentery, whereas only 225 Korean dysentery patients were recorded. In 1934, there were 4,113 patients with infectious diseases in the main cities of Korea; among them 2,789 were Japanese, although the number of Korean patients was probably underestimated.^45^ In addition to the practical necessity of controlling disease among Japanese personnel, there was an ideological imperative for sanitary intervention. When Japan expanded into neighbouring countries, it emphasised ‘the social superiority’ of its people by way of justification for imperial rule. Like Western imperial powers, the Japanese regarded the eradication or, at least, control of infectious diseases as a duty incumbent on any civilised nation or empire. Since the Meiji Restoration, Japan had whole heartedly embraced Western medicine, especially the laboratory-oriented medicine of imperial Germany.^46^ After the opening of Korea's ports, it tried to implant ‘civilisation’ in Korea with sanitary policies designed to control the indigenous population. In particular, Japan aimed to sanitize the capital Seoul and reform the hygienic habits of its residents and presented these efforts as part of its civilising mission.^47^ According to foreign critics, however, it was one of the chief faults of the public health organisation in Korea that the programme was Japanese directed, Japanese enforced and Japanese centred. The Koreans themselves had little part in the programme and little was done to improve their general health during the colonial period.^48^ Indeed, the sanitary police hardly paid any attention to the problem of malaria, even though many middle-aged Japanese residents were affected by the disease in Korea (Figure 1).

Most adult Japanese who came to Korea were exposed to endemic malaria for the first time, by contrast with most Koreans, who would have encountered malaria as a child. Like other persons raised in highly malarious areas, Koreans exhibited a degree of clinical resistance to malaria. It is well known that parasitemia prevalence rates are highest in childhood and early adolescence and decrease thereafter.^49^ This pattern accords with recent studies of age-related susceptibility to malaria among non-immune transmigrant people or travellers.^50^ In colonial Korea it continued to the mid-1920s. The differential incidence of malaria among Japanese and Korean populations can be seen from a study undertaken by the director of the Kangreung Provincial Hospital. Kangreung is located in Kangwon Province, in which malaria was severe. The director recorded that there were 8,606 Korean and 539 Japanese residents and collected the number of Japanese and Korean patients who were treated for malaria at the hospital. When doing so, he noted that Koreans were more likely to use traditional medicines and had ‘superstitious’ beliefs which deterred them from seeking treatment in hospital. The number of Koreans suffering from malaria was therefore likely to be underestimated. Nevertheless, the number of Korean patients visiting the hospital was large enough to show some interesting differences in the incidence of malaria among Koreans and Japanese, particularly the ages of the patients. He pointed out that 498 Japanese and 2,587 Korean patients were treated for malaria at the hospital from 1917 to 1925, the vast majority of the latter being below the age of 15.^51^ Indeed, until the 1920s, malaria was one of the principal causes of death among Korean children (Table 1).

**Table 1.** Causes of death among Korean children

	1 year	2–5 years	6–10 years	Total
‘Convulsions’	1841	2237	150	4228
Smallpox	81	1118	207	1406
Measles	47	466	102	615
Scarlet fever	15	55	102	83
Diphtheria	14	71	24	109
‘Fevers’	8	66	25	99
Cholera	–	42	29	71
Dysentery	27	473	137	637
Diarrhoea	22	114	18	154
Typhoid (or Typhus)	–	3	2	5
Cholera Nostras	–	8	1	9
‘Indigestion’	77	529	135	741
Malaria	28	401	76	505
Tuberculosis	1	19	7	27
Empyema	2	5	2	9
‘Colds’	39	188	39	266
‘Cough’	7	78	21	106

*Note*: 5,000 wives gave answers to the questions made by the author. 20,454 births were reported, and 9,325 deaths up to 10 years of age (Korean count).

*Source*: J. D. Van Buskirk, ‘Public Health Problems in Korea, as Shown by a Study of Child Mortality’, *The China Medical Journal*, 1927, 41–3, 244–50.

In contrast tp Koreans, the majority of Japanese malaria patients continued to be middle-aged. The susceptibility of this age group was problematic because it included key colonial personnel, including soldiers. In the Japanese garrisons in Korea, malaria was one of the chief nuisances and military doctors began to turn their attention to malaria as a specific problem rather than leaving it to be tackled by the sanitary police. From 1922 to 1925, the annual average number of malaria patients in Japanese garrisons was just above 250. Of the total of 1,037 malaria patients during this period, 732 were from the Hamheung Infantry, South Hamgyeong Province in the nineteenth division (the recurrence rate was 26 per cent). During the same period, the annual average number of malaria patients in the twentieth division was just above 150, and the total number of patients was 614, the largest number being from Daejeon, South Chungcheong Province (where the recurrence rate was 16 per cent). From 1908 to 1925, the average morbidity of malaria among soldiers of divisions in Japan was around 4 per thousand, whereas from 1917 to 1920 the average morbidity among Japanese soldiers in Korea was 55 per thousand, decreasing to 36 per thousand in 1925.^52^ One Japanese army officer pointed out that malaria had spread over the whole of Korea and many Koreans were suffering from this disease. However, only a few of them were cured completely; the rest ignored their symptoms or relied on the natural healing process. In addition, many Koreans slept outside during summer without mosquito nets. Such habits created a ‘reservoir’ of infection among Koreans to which Japanese soldiers were exposed, even though measures were taken to protect them inside the garrisons.^53^ Sanitary reform in the vicinity of military barracks therefore seemed to be an urgent necessity. The colonial government's inactivity in relation to malaria, according to some army officers, stood in marked contrast to its energetic measures against distoma pulmona (a parasitic infection caused by a species of lung fluke) and leprosy, which posed little or no danger to military effectiveness.^54^

From 1923 to 1931, the Government-General earmarked a sum of money for research on endemic diseases. During the first two years, these funds supported research on distoma pulmona as well as propaganda activities to let people know about the disease. Laws were also enacted to prevent the consumption of raw crabs, which was the main way in which the disease spread. The supply of information and the 1924 regulation forbidding the eating of raw crabmeat resulted in a decrease of pulmonary distoma.^55^ Although this disease presented a not insignificant health problem, and a potential cause of illness among troops, the amount of attention devoted to it may also be explained by the fact that Japanese researchers in Korea and Taiwan were taking the lead in elucidating this disease.^56^

Leprosy was privileged for different reasons. In 1916, the Government-General built a national leper sanatorium on Sorok island to demonstrate the colonial government's philanthropic activities to other countries.^57^ From the 1920s, the colonial government also began to support missionary leper sanatoriums in Busan, Gwangju and Daegu to control Western missionary groups operating there and to manage lepers in these localities using the agency of missionaries. From the 1930s, the Japanese royal family began to take an interest in this work, encouraged by the Japanese government which was keen to establish the royal family as the pivot of totalitarian rule. The family's philanthropic work was portrayed as an expression of its divine and merciful nature. Related to this, the colonial government of Korea organised the *Chosen Alliance to Defeat Leprosy* in 1932 and three years later the *Laws to Prevent Leprosy* (*Chōsen Rai Yobōrei*) were enacted. The colonial government collected contributions not only from civil servants but also citizens in its drive to enlarge the national sanatorium. This policy led to the deportation of lepers from other regions to the sanatorium; not only wandering lepers, but also some settled persons suffering from the disease.^58^

Morbidity and mortality from the two diseases mentioned above was far less than that from malaria. In 1920, distoma pulmonary patients amounted to 5,910 (Japanese 172, Koreans 5,734, foreigners 4) and the number of deaths was 1,443 (Japanese 19, Koreans 1423, foreigners 1). As far as leprosy was concerned, the number of patients was 2,604 (Japanese 20, Koreans 2,584) and of deaths, 321 (Japanese 1, Koreans 320).^59^ In the same year, 65,227 people (Japanese 6,107, Koreans 59,013, foreigners 107) were counted as malaria patients and 3,729 people (Japanese 38, Koreans 3,691) died from the disease. Due to the anti-distoma pulmonary measures, in 1927 cases of this disease decreased to 3,830 (Japanese 86, Koreans 3,743, foreigners 1) and deaths decreased to 922 (Japanese 4, Koreans 918). Even though it was difficult to determine the number of cases and deaths from malaria among Koreans, the number of recorded patients increased rapidly to 121,964 (Japanese 8,561, Koreans 113,143, foreigners 260) but deaths decreased to 2,084 (Japanese 26, Koreans 2,058).^60^

Harujirō Kobayashi, who was the director of the Government-General hospital, reported on the changing malaria situation in Korea at a meeting on the Interchange of Health Officers at the League of Nations, in November 1925:

Generally the extensive prevalence of malaria fever in Chosen is attributable to unsatisfactory prophylactic measures, especially insufficient use of mosquito-nets and the old habit of sleeping out of doors in summer. In this respect, however the persistent efforts of the health authorities to promote better-living conditions on the one hand, and the administration of quinine as remedy on the other, have brought about in recent years a remarkable diminution of patients.^61^

As he pointed out, the supply of quinine and knowledge of mosquito transmission had reduced mortality from malaria.^62^ However, while Dr Kobayshi took pride in the sanitary administration of the Government-General, the number of malaria cases had increased following annexation by the Japanese and the death toll often exceeded 2,000 (Table 2).

**Table 2.** Number of malaria patients and deaths from 1920 to 1927

	Patients				Deaths			
	Japanese	Korean	Foreigner	Total	Japanese	Korean	Foreigner	Total
1920	6,107	59,013	107	65,227	38	3,691	—	3,729
1921	5,996	61,458	167	67,621	33	2,896	2	2,931
1922	5,258	58,917	115	64,290	32	1,828	—	1,860
1923	5,026	57,339	227	62,592	32	1,388	—	1,420
1924	5,044	63,290	170	68,504	16	1,821	—	1,837
1925	7,043	91,939	151	99,133	23	2,021	—	2,044
1926	7,427	107,286	302	115,015	19	2,384	—	2,403
1927	8,561	113,143	260	121,964	26	2,058	—	2,084

*Source*: Chōsen Sōtokufu Keimukyoku, *Chōsen Bōeki Tōkei 1934* (Seoul: Chōsen Sōtokufu, 1935), 135.

This increase in malaria cases seemed to be linked to the Government-General's economic policies: particularly, the creation of government-controlled salt-farms and the promotion of irrigation to increase rice production. The population was increasing but the percentage of malaria cases per population also increased (Table 3).

**Table 3.** Malaria cases and populations in Korea

Year	Total	Japanese	Korean	Foreigners
1920	17,288,989	347,850 (1.76%)	16,916,078 (0.35%)	25,061 (0.43%)
1922	17,626,761	386,493 (1.37%)	17,208,139 (0.34%)	32,129 (0.36%)
1924	18,068,116	411,595 (1.23%)	17,619,540 (0.36%)	36,981 (0.46%)
1926	19,103,900	442,326 (1.7%)	18,615,033 (0.56%)	46,541 (0.65%)
1928	19,189,699	469,043 (2.2%)	18,667,334 (0.75%)	53,322 (0.75%)

*Note*: the percentage of cases per population is shown in parentheses.

*Source*: From each year of *Chōsen Bōeki Tōkei* (Seoul: Chōsen Sōtokufu).

From 1907 to 1920, government-controlled salt farms were constructed in Kyeonggi Province and South Pyeongan Province to compete with cheap Chinese salt and acquire revenue from sales.^63^ The construction of these farms led to workers becoming infected with many diseases. In 1918, the staff and families of a branch office of the monopoly bureau located near the salt farm constructed in South Pyeongan Province were also suffering from malaria and typhoid fever.^64^ During the early 1920s, while a salt farm was under construction in Kyeonggi Province, many workers were affected by diseases (including malaria) and physical fatigue. In addition, sewage and drainage systems were incomplete. Thus people who lived around the construction often suffered from flooding. As a result, in 1926, the monopoly bureau of the Government-General promised to build drainage ditches in this area.^65^

To improve the supply of rice to Japan, the Government-General began at the same time to increase rice production, the so-called *Sanmi Jeungsan Gyehoek*. In 1918, due to the inflated price of rice, riots occurred all over Japan. In response, Japan put into operation plans to secure national self-sufficiency in foodstuffs and the Government-General of Korea supported it. The substance of the plan was the building of irrigation systems, reservoirs and land reclamation. In 1926, the Irrigation and Drainage and the Reclamation Divisions were founded by the government and in December of the same year the government enacted the Chosen Irrigation Association Law to prompt construction of irrigation systems. The irrigation rate (proportion of agricultural land irrigated) increased from 24.5 per cent in 1918 to 35.1 per cent in 1925, and from 1935 the irrigation rate increased to more than 47 per cent.^66^ However, the fund was formed from revenue taken from tenant-farmers (mostly Koreans) and these policies led to a depression in agriculture which left many farmers ruined. Also, Korean agriculture depended increasingly on the rice-field farm system.^67^ Its reservoirs and irrigation systems provided an ideal breeding environment for mosquitoes. The main breeding areas for *Anopheles* in Korea were paddy fields, ditches, puddles, and even sewage and water plants.^68^ These changes and their consequences were in many respects similar to those which occurred in Taiwan under Japanese rule. During the late nineteenth and early twentieth century, the cultivation of rice and sugar farms, which depended on the introduction of new year-round irrigation technology, led to many severe epidemics of malaria.^69^

Another contributing factor to the rise of malaria in Korea during the 1920s was the influx of Chinese migrant labourers, particularly from the Shandong peninsula which was one of the most malarious areas of China.^70^ In 1920, the Chinese population of Korea was 23,989 and five years later it increased to 58,057. On the other hand, the number of foreigners from other countries was only 1,264.^71^ The Chinese settled all over the country but about 43 per cent lived in North Pyeongan and Kyeonggi Provinces. They were engaged in reclamation works, civil engineering, farm work, transportation and so on.^72^ Many Chinese labourers were working on the construction of salt farms in Kyeonggi, which left them exposed to diseases; their residential areas were also very unhealthy.^73^ Kyeonggi Province was one of the provinces most severely affected by malaria and in 1928 there were a total of 29,348 malaria patients, 1,148 of whom were counted in the city where the salt farm was located.^74^

There was also an influx of Korean and Japanese labourers into the port of Busan during the 1920s. Because of the decline of agriculture, the development of industries, construction works for the harbour and so on, Busan experienced a wave of immigration and this led to housing problems and the enlargement of slum areas in the city. Poor water supply and sewerage worsened the sanitary environment.^75^ In the early 1900s, it was said that malaria had almost disappeared from Busan.^76^ However, in 1921, there were 268 Japanese and 139 Korean malaria patients, the number of cases among Koreans probably being underestimated.^77^ Although sanitary reforms were undertaken energetically from the 1900s, the infrastructure was concentrated in the former Japanese settlement and annexed new town. The slum areas and Korean villages were untouched by sanitary reform and remained vulnerable to infection.

As mentioned above, Kobayashi and other Japanese doctors stressed that malaria was to be found all over the country, whether in rice fields or mountainous areas. They drew attention away from the changes made by the Japanese and emphasised instead the ‘uncivilised habits’ of Koreans, such as sleeping out of doors, their failure to use mosquito nets and their dependence on ineffective and superstitious remedies.^78^ They said nothing of poverty. In other countries, too, malaria tended to thrive among poor farmers who were displaced from the land or who retained a small plot of land but lacked the resources to improve it. Such populations often lived in inadequate shelters and subsisted on nutritionally poor diets.^79^ For example, Margaret Jones has described the findings of the WHO entomologist DrVan Seventer who visited Jamaica in 1960 and who concluded that the persistence of malaria in the parish of Clarendon was caused by people sitting outside in the evenings during the anopheles' biting hours. The reason for this obviously risky behaviour was that their houses were small and could be occupied only for sleeping.^80^ Malaria also tended to affect whole families at the same time.^81^ This trend was related to susceptibility to other diseases. In Ceylon, for example, infection with malaria affected the delivery of food and water, often leading to infection with other diseases.^82^ Moreover, *P. vivax* which was common in Korea recurs easily because the *vivax* parasite can reside in the liver for lengthy periods of time before re-emerging to reignite malaria symptoms. *P. vivax* was a great debilitator, rather than a great killer, although its capacity to kill and cause serious illness (especially among children and pregnant and lactating women) is often underestimated.^83^ Many Koreans were unable to afford proper treatment for malaria and their health was additionally threatened by their poor diet and insanitary living conditions. This accords with J. R. McNeill's finding that with the inauguration of a ‘golden age’ of health around 1885–1920, differential protection against infectious disease increasingly correlated with the wealth of the society in which one lived, rather than with one's personal prior disease experience or that of one's ancestors.^84^ The Japanese doctors and the Government-General did not invest in the prevention of malaria until 1927 and persisted in blaming the disease on Koreans.

## Malarial fluctuations

Just after the annexation, Takeuchi, an army medical officer, reported on the general sanitary condition of Korean society. He drew attention to the problem of malaria among Japanese civilians and Koreans as well as Japanese soldiers; however his main focus remained on malaria in Japanese garrisons.^85^ The largest number of malaria patients came from the infantry division in Hamheung and the situation appeared to be getting worse. In 1922, 176 patients were treated for malaria but in 1926 the figure increased to 259. Thus, in 1926, this division began to take active anti-malaria measures, such as spraying oil into ditches and puddles, using mosquito repellents and nets. It also supplied quinine to soldiers as a prophylactic. These measures—which were carried out at the behest of the army and implemented by its personnel—appear to have produced a slight decline in the number of patients. In 1925, the number of patients was 274 and it decreased to 259 in 1926.^86^ From 1928, this division isolated malaria patients and introduced systematic treatment and mosquito eradication.^87^

Unlike Japanese military officers, the Government-General did not begin to support research into malaria until the summer of 1928. In each colonial settlement, there tended to be a different hierarchy of disease problems. Although the Japanese settlers were suffering badly from several infectious diseases, their mortality from malaria was low (Table 4).

**Table 4.** Number of patients and deaths from main infectious diseases and malaria in 1940

	Patients				Deaths			
	Japanese	Korean	Foreigner	Total	Japanese	Korean	Foreigner	Total
Typhoid	2,310	9,777	14	12,101	320	1,474	2	1,796
Dysentery	2,581	1,579	8	4,168	365	389	—	754
Diphtheria	992	1,725	6	2,723	76	479	3	558
Smallpox	135	3,128	2	3,265	18	611	—	629
Malaria	4,336	88,417	331	93,084	44	965	12	1,021

*Source*: Chōsen Sōtokufu Keimukyoku, *Chōsen Bōeki Tōkei 1940* (Seoul: Chōsen Sōtokufu, 1942), 2–3, 284–285.

In view of this, the colonial government's sanitary policies continued to concentrate on infectious diseases. A few Japanese medical officers and Korean doctors did do research on malaria but the central government did not distribute free or reduced price quinine to civilians, although a few local governments provided it for civilians, on their own initiative, when a severe epidemic of malaria occurred. In July 1928, in South Pyeongan Province, which was still severely affected by malaria, the local government distributed quinine free of charge to the inhabitants.^88^ In the next month, the board of health of the provincial office began to investigate carriers of plasmodium.^89^ Anti-malaria measures also commenced in South Chungcheong Province, which was also badly affected by malaria, including the quartan type which was uncommon in Korea.^90^ The Daejeon garrison of the twentieth division, which had one of the largest number of military malaria patients, was located in this province, so the control of malaria could not be ignored. The board of health of this provincial office surveyed the carriers of plasmodium and there were about 15,000 people, normally under the age of twenty, who had the parasite. This office provided enough quinine for 7,000 people, but the amount was insufficient.^91^

Reviewing the trend of hospital admissions and deaths from malaria, in 1928, when malaria research began, the number of patients and deaths actually increased from 121,964 to 151,389 and from 2,084 to 2,417. But Japanese deaths from malaria decreased from 26 to 23; by contrast, the deaths of Koreans increased from 2,058 to 2,394 (Table 5). But from 1930, except for 1934, the patients and deaths from malaria decreased overall with a few exceptions.

**Table 5.** Number of malaria patients and deaths, 1928–1940

Year	Patients				Deaths			
	Japanese	Korean	Foreigner	Total	Japanese	Korean	Foreigner	Total
1928	10,566	140,426	397	151,389	23	2,394	—	2,417
1929	11,513	154,608	470	166,591	27	2,139	2	2,168
1930	9,836	145,045	306	155,187	16	1,718	1	1,735
1931	8,499	130,153	291	138,943	21	1,562	—	1,583
1932	6,437	127,580	177	134,194	21	1,786	1	1,808
1933	6,510	119,626	182	126,318	24	1,646	1	1,671
1934	6,244	140,012	113	146,369	13	1,752	1	1,766
1935	5,713	125,468	213	131,434(*sic*)	11	1,194	1	1,206
1936	5,003	110,341	195	115,539	27	1,078	—	1,105
1937	4,422	101,565	167	106,184(*sic*)	13	1,117	—	1,130
1938	4,123	95,418	170	99,711	24	934	1	959
1939	4,378	92,622	223	97,223	9	1,040	3	1,052
1940	4,336	88,417	331	93,084	44	956	12	1,021

*Note*: The numbers of patients with and deaths from malaria were counted until 1940 to be convenient for duties of the Police Affairs Division.

*Source*: Chōsen Sōtokufu Keimukyoku, *Chōsen Bōeki Tōkei 1940* (Seoul: Chōsen Sōtokufu, 1942), 284–5.

In 1934, most parts of Korea, particularly in the south (Kyeongsang, Chungcheong and Jeolla Provinces) were damaged by severe flooding and frost. Heavy rainfall and flooding badly affected the harvest.^92^ Among the damaged areas, Kangwon, Pyeongan and Hamgyeong Province in North Korea were hardest hit and in these areas 190,000 people were suffering from famine.^93^ Nevertheless, in this year, Korea exported increasing amounts of rice to Japan, Kwantung (as military supplies) and Manchuria, for the South Manchuria Railway.^94^ Taken together, these rapid increases in population, rice production and exportation caused rice consumption in Korea to fall from 198 lbs. per capita per annum in 1916–20 to 119 lbs in 1931–35.^95^ Due to flood damage and continuing famine, many people lost their homes and jobs, causing them to move in search of food and work; many farmers consequently left their home towns and stayed in improvised shelters.^96^ These conditions produced a dramatic increase in cases of malaria. For example, during the summer of 1934, victims of flooding in Kyeongsang Province were attacked by malaria and dysentery and a relief squad was dispatched to the damaged areas.^97^ Malaria patients of Siheung, where the government-controlled salt farm was located, increased from 487 to 678.^98^ From the most severely flood-damaged Provinces—Chungcheong, Jeolla and Kyeongsang—61,912 patients and 733 deaths were recorded. And 50,803 patients and 270 deaths, all Koreans, were recorded in the areas of North Korea which experienced a bad harvest.^99^ In these provinces, many people practised fire-field farming (slash-and-burn) and their insanitary living conditions and poor nutrition caused many diseases. As a result, mortality in these areas was higher than other provinces.^100^

In the following year, the malaria epidemic abated and the number of patients and deaths decreased steadily. In 1935, most of the provinces showed a fall in malaria patients and deaths, except South Chungcheong Province. One of the reasons why the disease decreased seems to be the drier weather. At this time, many areas were suffering from drought throughout the year.^101^ Kyeonggi Province, which included Seoul, Incheon, Siheung and other agricultural areas, recorded a rapid decrease in patients and deaths—albeit unevenly. Overall, the number of patients fell from 14,478 to 9,198 and deaths decreased from 584 to 370.^102^ But in Seoul and Incheon, malaria patients increased from 1,308 to 1,458 and from 152 to 550.^103^

In general, there is a positive correlation between urbanisation and the decline of mortality and morbidity from malaria, chiefly because it tends to reduce opportunities for *Anopheles* to breed.^104^ However, this trend is by no means universal, as has been shown by the periodic resurgence of malaria in many Asian and African cities since the 1970s. We can observe historical parallels for these fluctuations in the experience of Seoul and Incheon between 1928 and 1936; cities which showed an increase in malaria at a time when they were undergoing rapid development (Table 6).

**Table 6.** Malaria patients in Seoul and Incheon of Kyeonggi Province, 1928–1936

	1928	1929	1930	1931	1932	1933	1934	1935	1936
Seoul	1,691 (46)	2,238 (22)	1,528 (22)	1,440 (14)	1,016	1,876 (13)	1,308 (9)	1,458 (7)	1,341 (5)
Incheon	249 (3)	230 (16)	63 (5)	85 (6)	120 (8)	116 (15)	152	550 (5)	116
Kyeonggi	29,348	28,021	23,480	18,983	16,770	17,799	14,478	9,085	6,114
Province	(59)	(87)	(55)	(35)	(10)	(45)	(30)	(113)	(9)

*Note*: the number of foreigners is given in parentheses.

*Source*: Chōsen Sōtokufu Keimukyoku, *Chōsen Bōeki Tōkei 1934–36*; Kyeonggidō Eiseika, *Eisei Kaiyō* (Kyeonggidō: Kyeonggidō Eiseika, 1937), 174.

One reason for this increase in urban malaria may have been the fluidity of the population in these cities, a large proportion of which was made up of labourers. During the 1930s, the Government-General planned to absorb surplus labour from rural areas by employing men in the defence industries under the quasi state of war since the Japanese invasion of Manchuria.^105^ Many men went to the cities but could not get a proper job. As a result, they became fire-field farmers or casual labourers; many remained jobless for much of the time and most lived in insanitary conditions.^106^ Thus, the rate of poverty and mortality in the big cities such as Seoul and Incheon was higher than other small cities which had relatively good hygiene conditions.^107^ Seoul, the capital city of colonial Korea, was a notorious hot-bed of disease. In 1935, incidences of malaria were rising there, unlike in other provinces, while typhoid fever and dysentery were also severe.^108^ Generally, cases of epidemic disease per 10,000 people in Seoul were 62.8, in contrast to levels around 25 in big cities in Japan.^109^

Among the labourers in Seoul, there were two carriers of the *P. falciparum* parasite, hitherto unknown in Korea. Discovered in 1936, these labourers had been to Osaka and other places in order to find work. They also had a history of several diseases such as measles and typhoid fever. Also, both were affected by malaria during childhood.^110^ Including one *P. falciparum* case found in Hwanghae Province, ten carriers of this parasite were recorded in Korea up to 1939.^111^ Instances of *P. falciparum* malaria acquired in Korea were confined to drug addicts who contracted the disease through inoculation with improperly sterilized hypodermic needles.^112^

Although exceptional malaria epidemic conditions had occurred in Korea, the number of cases and deaths from malaria was decreasing gradually because of interventions by a few provincial governments. In addition, through various measures, Korean rice production was raised from an annual 1.81 million metric tons in 1916–20 to 2.73 metric tons in 1936–40 following the *Sanmi Jeungsan Gyehoek*. Although this Japanese policy proved dietetically harmful to the Koreans, it modified the economic status of Korean peasants by giving them a cash income, incidentally increasing demand for Japanese manufactures of non-food items. By 1936, Japan, having completed her programme of self-sufficiency in food stuffs, showed less inclination to exploit Korean rice resources, although exports to Japan were still large enough to cause a serious famine when the crops failed in 1939, with the result that rice consumption in Korea rose slightly to 152 lbs, per capita per annum in 1936–40.^113^ This meant that some Koreans had a slightly improved nutritional status and were more able to resist infection. It also meant that they were better able to prevent infection, for example, by being able to purchase quinine and medical treatment. But this was an unstable situation. When the crops failed because of severe drought in 1939, deaths from malaria among Koreans increased again, most likely due to famine.^114^

Furthermore, urbanisation and the development of remote parts of the country had been increasing steadily. For example, in 1932–33, the government earmarked a budget of ¥4,255,153 (accounting for more than half of the increase in total expenditure that year), for the development of North Korea. These plans could hardly fail to have had military importance. The *Sanmi Jeungsan Gyehoek*, the attempt to develop north-east Korea and to rescue it from the wasteful and destructive ‘fire-field’ methods of the past, was also making progress, although it was considerably hampered by a lack of funds following government retrenchment.^115^ Drainage systems were extended during the 1930s, even though most expenditure on construction was met by levying a fee on farmers for irrigation. In 1931, the irrigation association of Gunsan, in North Jeolla Province, planned to complete drainage systems to prevent flood damage.^116^ In South Hamkyeong, from 1936 agricultural drainage systems were established and existing drainage systems were repaired.^117^

In these ways, the epidemic situation was gradually relieved. However, mortality from malaria remained much higher among Koreans than Japanese. From 1928, the number of deaths among Japanese was fewer than 30, with the exception of 1940. During this period, the average mortality among Japanese was 0.31 per cent, while the average mortality among Koreans was 1.23 per cent. Even if the number of Korean patients was underestimated, the average mortality was still four times higher than among the Japanese. This reflected the different economic situation of the two populations and their differential access to medical facilities. The total number of Japanese patients treated in government hospitals in Korea during 1938 was 334,438, while the number of Korean patients was only 389,739—a tiny proportion of the population as a whole. In the same year, the Japanese population was 633,522, whereas the Korean population was 21,950,616. Assuming that each patient was treated only once, it could be inferred that one in two Japanese and one in 56 Koreans was treated in these hospitals.^118^ In the same year, 149 hospitals were counted in Korea, and there were approximately 13,800 beds. Of these, however, around 6,900 were in leper colonies, and about 400 beds were in military and naval hospitals. Excluding these beds, Korean hospitals provided only approximately 2.7 beds per 10,000 people as compared to an average of 34 per 10,000 people in Japan, and 100 beds per 10,000 people in the USA.^119^ It appears, therefore, that the hospitals primarily served the Japanese, while the Koreans received far less attention. Even though the Government-General began to pay attention to Korean traditional medicine from the 1930s—in order to make up for the shortage of modern hospitals—this expedient did not work. Throughout the colonial period, many Koreans, particularly those in rural areas, found it difficult to access either traditional medical services or Western-style dispensaries.^120^

Apart from the lack of medical facilities for Koreans, their general condition was poor. The life of a tenant farmer was penurious, with stiff rents paid in kind and general insecurity of tenure.^121^ Moreover, in 1938, the Japanese government enacted the *National General Mobilisation Act* to control human and material resources for the war. This movement affected Korea, too. The Government-General enacted the Wage Control Ordinance and the Regulation of Working and under these regulations many labourers were exploited.^122^ Enormous human and material resources were also extracted from Korea to aid the Japanese war effort. Faced with the worsening economic situation and a deteriorating medical environment, Koreans found it much harder to access modern medical treatment than the Japanese, with the result that mortality from malaria among Koreans remained high. To be successful, malaria control programmes need to be an integral part of a well-organised health service, bolstered by improvements in standards of living.^123^ This happened in Korea to some extent under Japanese rule but the improvement was uneven and fitful due to fundamental inequalities between health care and living standards for Koreans and Japanese.

From 1941, the Government-General stopped collecting statistics relating to malaria and it is therefore impossible to gauge accurately the impact of wartime conditions on the malaria situation in Korea. However, qualitative evidence suggests that after 1940 malaria once again severely affected Korean society. According to the US military, malaria was increasing during the last years of the war. Thus malaria, as well as venereal diseases and dysentery, would be of great concern to the occupying forces after Korea's occupation by the American Army, the report warned.^124^ However, just as the Japanese colonisers had neglected malaria among civilians in Korea, this neglect continued during the Korean War of 1950–53. One Canadian medical doctor was especially critical of this, nothing that while the malarial situation in Korea was well known prior to the war, the United Nations forces had done little to prepare themselves.^125^

One of the issues leading to the malaria problems of the post-war period had been the shortage of quinine during the Second World War.^126^ In the colonial period as a whole, about 99 per cent of all drugs and hospital supplies were imported from Japan.^127^ After the Second Sino-Japanese War broke out, malaria patients among Japanese soldiers increased rapidly because of fighting in malarious areas in southern China. In addition, the importation of cinchona, and of synthetic anti-malaria drugs such as atebrin and plasmochin, became difficult for Japan after the USA revoked the Treaty of Commerce and Navigation between Japan and the USA in 1940.^128^ The Japanese navy defeated the Allied task force under the Dutch rear admiral Doorman at the Battle of the Java Sea, 27–28 February 1942, and the Allies were cut off from Dutch quinine and rubber supplies: both strategic commodities.^129^ The invasion of Java eased the shortage of quinine temporarily, but as the battle fronts extended, the shortage of anti-malaria drugs among Japanese solders worsened and became common in other parts of the Japanese empire as well. For example, in 1940, the Fushun mining operations of the South Manchuria Railway company were also falling short of anti-malarial drugs.^130^ If these economic and strategic assets were deprived of malaria treatments, there was little hope for Korean civilians. After 1940, all resources, such as they were, had to be concentrated not on Korea but on personnel fighting against the Allied Forces and the Chinese.^131^

## Conclusion

Before Korea became subject to Japanese control, malaria was often recorded in the country. The disease existed as a daily menace, although it predominantly affected children. After the opening of the ports in 1876, malaria began to threaten foreigners, too. Throughout the port opening period, the Japanese began to be badly affected by the disease, the majority of cases occurring among adults who had not acquired immunity in their childhood. Consequently, malaria undermined Japanese military and economic power. However, for some time after the annexation of Korea in 1910, except for a few investigations of malaria and anti-malarial work in Japanese garrisons, the Government-General did not take any special measures against malaria, even for Japanese civilians. It did not want to accept the financial burden such policies would entail and it principally focused on infectious diseases which caused higher mortality among the Japanese. The Japanese authorities knew that malaria control on a large scale would be an expensive undertaking. In Taiwan, where the malaria problem was worse than in Korea, they also sought a short-term and relatively cost-effective solution in the use of quinine, concentrating on the ‘human factor’ in malaria rather than environmental reforms.^132^ The Government-General of Korea did not begin to investigate the malaria situation until 1927, with the exception of the malaria problems in Japanese garrisons. The Japanese authorities simply criticised the Koreans' ‘uncivilised manners’ and ignored their impoverished situation.

From 1928, however, the government began to organise research on malaria and a few local governments themselves took anti-malaria measures. These limited interventions did nothing to improve the situation and, until the end of 1920s, cases and deaths from malaria continued to increase. Whatever beneficial effects these programmes had, they were outweighed by the surge in malaria caused by the government's economic policies. With insufficient drainage, economic development made the malaria situation worse.

During the 1930s, the incidence of malaria in Korea's largest cities—such as Incheon and Seoul—remained unstable because of an influx of labourers. But, in Korea as a whole, malaria cases and deaths began to decrease, with a few exceptions. an increase in 1934 was due to flood damage and insanitary and unhealthy living conditions. Again, inadequate drainage systems aggravated the situation. Subsequently, drainage systems were extended to deal with floods, while general development policies seemed to contribute to the reduction of malaria transmission. But these policies were not taken specifically to deal with malaria and some were designed to assist the war effort after the Japanese invasion of Manchuria. Also, the funds for these projects was acquired from the Korean people, causing further financial hardship.

After the Second Sino-Japanese War broke out, along with the *National General Mobilisation Act* in 1938, the burden of malaria on Koreans became even heavier. The mortality of Koreans was much higher than that of Japanese due to the difficulties of accessing modern medical facilities and economic hardship. In addition, lack of goods including medicine became more serious under the war regime. Quinine was unavailable and malaria once again severely affected Korean society. Moreover, the *vivax* parasite developed the ability to ‘winter over’ inside its human host. During the following warm season, the parasite circulating in the human blood could then infect the next generations of vector mosquitoes and the cycle would be repeated.^133^ Continuing treatment was therefore the best way to save people's lives but many Koreans could not afford to keep up their treatment for a sufficient length of time. By exhausting its victims, and rendering them incapable of work, malaria also worsened such painful situations as existed, its impact being felt far beyond the number of recorded patients and deaths from this disease.
